# Mechanical interaction between sequential Micra devices resolved by programming alone: a case of premature battery depletion in a 91-year-old patient

**DOI:** 10.1093/ehjcr/ytag431

**Published:** 2026-06-15

**Authors:** Changjian He, Tianze Guo, Chunhua Ding, Wenchang Zhang

**Affiliations:** Cardiac Department, Aerospace Center Hospital, Peking University Aerospace School of Clinical Medicine, 15 Yuquan Road, Haidian District, Beijing 100049, China; Cardiac Department, Aerospace Center Hospital, Peking University Aerospace School of Clinical Medicine, 15 Yuquan Road, Haidian District, Beijing 100049, China; Cardiac Department, Aerospace Center Hospital, Peking University Aerospace School of Clinical Medicine, 15 Yuquan Road, Haidian District, Beijing 100049, China; Cardiac Department, Aerospace Center Hospital, Peking University Aerospace School of Clinical Medicine, 15 Yuquan Road, Haidian District, Beijing 100049, China

**Keywords:** Leadless pacemaker, Micra AV, Oversensing, Device–device interaction, Battery depletion, Programming

## Abstract

**Background:**

The Micra transcatheter pacing system provides a leadless alternative for patients requiring ventricular pacing. When a leadless device reaches end of service or fails prematurely, a second device is commonly implanted.

**Case summary:**

We report a 91-year-old man with premature Micra AV battery depletion 28 months after implantation. During the second implantation, mechanically mediated device–device interaction caused intermittent oversensing. Oversensing was resolved by increasing the sensing threshold, which avoided device repositioning or retrieval.

**Discussion:**

Suspected mechanically mediated interaction between sequential Micra devices may cause oversensing and pacing inhibition. In patients with clinically stable condition and adequate intrinsic R-wave amplitude, programming optimization may be considered before invasive revision; however, future management should account for cumulative right ventricular device burden.

Learning points

- Sequential Micra devices can mechanically interact and cause oversensing with pacing inhibition.
- If intrinsic R-wave amplitude is adequate, increasing the sensing threshold may suppress mechanical artefacts and avoid invasive revision.

## Introduction

Leadless pacemakers, such as the Micra transcatheter pacing system (Medtronic, Minneapolis, MN), are established alternatives to transvenous pacing in selected patients. When a leadless pacemaker reaches end of service or fails prematurely, retrieval may be difficult because of encapsulation, and a second device is commonly implanted adjacent to the abandoned unit.^1–5^ Although the right ventricle can usually accommodate more than one Micra device, close proximity may lead to mechanically mediated device–device interaction and inappropriate sensing.^2,6^ We report a case of premature Micra battery depletion in which device–device interaction was resolved by programming alone.

**Figure 1:**
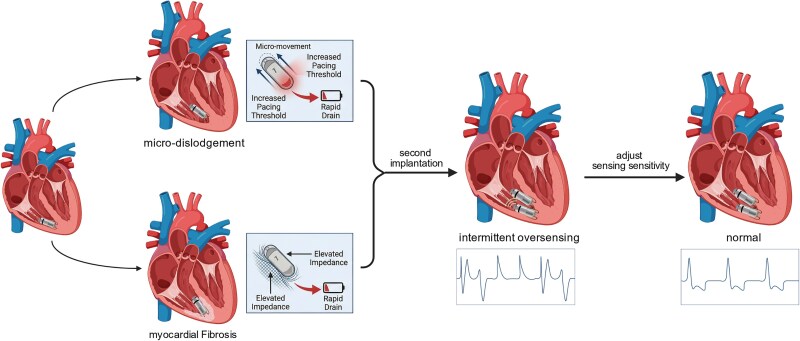
Sequential Micra implantation, suspected mechanically mediated device–device interaction, and successful resolution by programming adjustment.

## Case presentation

A 91-year-old man (body weight, 50 kg) with stage 3 hypertension underwent Micra AV implantation on 6 January 2023, for symptomatic third-degree atrioventricular (AV) block. Comorbidities included a small pericardial effusion and chronic pulmonary disease. Initial device parameters were satisfactory.

Approximately 27 months later, interrogation revealed premature depletion of the original Micra AV battery. The pacing threshold had increased to 3.0 V at 0.24 ms, impedance was 440 Ω, and the intrinsic R-wave amplitude was 7.4 mV. At recognition of premature depletion, the patient remained effectively 100% ventricularly paced, including 75% AV synchronous pacing (AM-VP) and 25% ventricular-only pacing. The patient’s advanced age and frail status resulted in the abandonment of the original device, and a second Micra was implanted.^4,5^

During the procedure on 6 May 2025, right ventricular (RV) angiography confirmed that the original Micra device remained positioned on the septum, with subtle micro-movement synchronous with systole (*Figure 2*). An inferior septal landing site was attempted but abandoned because the delivery system lacked adequate support for stable deployment. Therefore, a more superior septal position was selected, and a second Micra device was implanted successfully with good stability on tug testing (*Figure 3A* and *B*).

**Figure 2:**
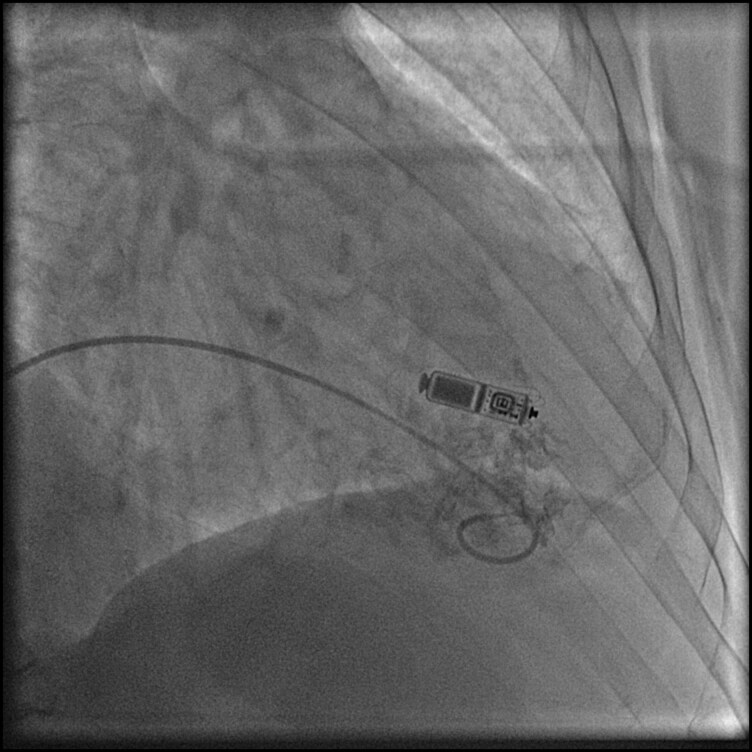
Initial angiography. Stable position of the original Micra AV device in the right ventricular septum.

**Figure 3:**
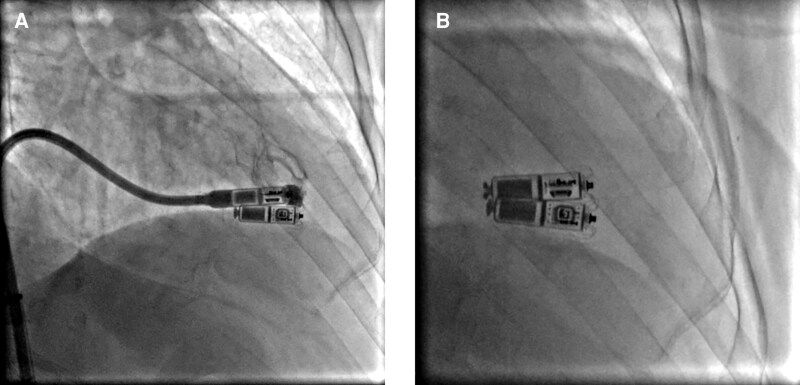
Second Micra AV implantation. (*A*) Superior septal deployment of the second Micra AV device. (*B*) Tug test confirming device stability.

Immediately after deployment, intermittent oversensing occurred, consistent with device–device interaction between the adjacent capsules (*Figure 4*).^6^ Increasing the sensing threshold to 2.0 mV eliminated oversensing and avoided repositioning. Final electrical parameters of the second Micra device were satisfactory: threshold of 1.0 V at 0.24 ms, R-wave amplitude of 5.1 mV, and impedance of 730 Ω.

**Figure 4:**
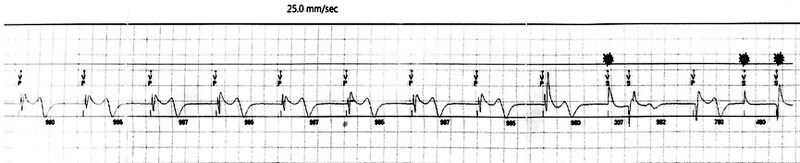
Intraoperative oversensing. Intermittent oversensing after second Micra device implantation, consistent with suspected mechanically mediated device–device interaction.

At 1-month follow-up, AM-VP accounted for 82.9% of ventricular pacing, with no recurrence of oversensing. At extended follow-up on 12 March 2026, the second Micra remained stable, with battery voltage of 3.01 V, estimated remaining longevity of 7.5 years, threshold of 0.75 V at 0.24 ms, impedance of 480 Ω, intrinsic R-wave amplitude of 8.3 mV, and sensing threshold of 2.0 mV. AM-VP accounted for 84.6% of paced beats, with no recurrent mechanical oversensing. Available device interrogation data at key time points are summarized in *Table 1*. No temporary pacing lead was implanted because the original Micra remained capable of providing ventricular pacing support during the procedure.

**Table 1:** Available device interrogation data at key time points

Time point	Device	Threshold	Impedance (Ω)	R-wave amplitude (mV)	Sensing setting (mV)	Pacing burden/synchrony	Battery status
6 January 2023 (initial implantation)	First Micra AV	0.88 V @ 0.24 ms	680	7.0	NA	NA	Normal
∼27 months after implantation, at recognition of premature depletion	First Micra AV	3.0 V @ 0.24 ms	440	7.4	NA	VP 100%; AM-VP 75%; VP-only 25%	Premature depletion recognized
6 May 2025 (second implantation)	Second Micra AV	1.0 V @ 0.24 ms	730	5.1	2.0	NA	New implant
12 March 2026 (extended follow-up)	Second Micra AV	0.75 V @ 0.24 ms	480	8.3	2.0	AM-VP 84.6%; VP-only 15.3%; AM-VS <0.1%; VS-only 0.1%	Battery voltage 3.01 V; estimated longevity 7.5 years

Historical memory of the original Micra device was limited to approximately 80 weeks; therefore, complete longitudinal trends in threshold, output settings, and battery voltage were not retrievable at later reinterrogation.

AM-VP, atrial mechanical event–ventricular paced; AM-VS, atrial mechanical event–ventricular sensed; AV, atrioventricular; Micra AV, leadless ventricular pacemaker with atrioventricular synchrony algorithm; NA, not available; VP, ventricular paced; VP-only, ventricular pacing without atrial mechanical tracking; VS-only, ventricular sensed without atrial mechanical tracking.

## Discussion

This case highlights two practical challenges in leadless pacemaker life-cycle management: premature battery depletion and suspected mechanically mediated device–device interaction after sequential Micra implantation. Battery depletion within 28 months after implantation is unusual. Real-world data support long battery longevity for Micra systems, and registry follow-up suggests that system revisions are more often related to threshold elevation than to primary battery failure.^1,7^ In this patient, premature depletion was recognized approximately 27 months after implantation when the ventricular pacing burden was 100%, including 75% AV synchronous pacing (AM-VP) and 25% ventricular-only pacing. Despite the unavailability of complete longitudinal output setting and battery-voltage trends because the device retained only the previous 80 weeks of historical data, threshold elevation had already developed within the retrievable interval. These findings (*Table 1*) support the likelihood that sustained high pacing burden together with progressive threshold rise contributed to accelerated battery drain; however, an intrinsic device-related mechanism cannot be excluded. Similar threshold elevation related to myocardial interface changes was also reported in other pacing systems.^8^

The proposed mechanism of oversensing should be interpreted cautiously. Direct proof of device–device mechanical contact was unavailable. However, the overall findings were most consistent with mechanically mediated device–device interaction based on the close fluoroscopic proximity of the two capsules, temporal association with systole, nonphysiological high-frequency signals distinct from intrinsic ventricular electrograms, and immediate disappearance of oversensing after reprogramming. Mechanical contact during systole can generate artefacts that fall within the sensing bandwidth of Micra AV systems and may be misclassified as intrinsic ventricular activity, resulting in pacing inhibition.^6^ Far-field atrial signals and myopotentials were considered less likely because the oversensing was temporally linked to the presumed device interaction and resolved promptly after adjustment of the sensing threshold.

Programming optimization was implemented because of the frail status of the patient, the original device remained capable of backup pacing, and the intrinsic R-wave amplitude provided a sufficient margin for safe adjustment. The sensing threshold was set to 2.0 mV because the intrinsic R-wave amplitude remained high (≥5.1 mV). In practice, programming the sensing threshold at approximately one-third to one-half of the intrinsic R-wave amplitude may provide an acceptable sensing margin while filtering mechanical artefacts. The main safety concern of this strategy is undersensing of true ventricular activity; therefore, it should only be considered when intrinsic R-wave amplitudes are stable and sufficiently large. In this case, the adequacy of the strategy was supported at the extended follow-up when the R-wave amplitude remained 8.3 mV with no recurrence of oversensing.

This case does not imply that programming should replace invasive revision in all cases of leadless pacemaker interaction. Extraction, repositioning, or alternative pacing strategies should still be considered when oversensing persists despite programming, intrinsic R-wave amplitudes are low or unstable, pacing inhibition causes clinical instability, or device positioning poses unacceptable mechanical risk. In pacemaker-dependent cases, backup pacing should be available. The present case did not require temporary pacing lead or asynchronous mode because the original Micra remained functional and could provide ventricular pacing support during the procedure.

The decision to abandon rather than retrieve the original Micra device was individualized. Abandonment is commonly considered when chronic encapsulation, advanced age, frailty, or procedural risk make retrieval less favorable.^4,5^ Nevertheless, abandoned leadless pacemakers occupy finite RV ‘real estate’. Preclinical and clinical reports suggest that multiple Micra devices can coexist in the right ventricle^9–12^; however, cumulative device burden may complicate future implantation, increase the likelihood of device proximity, and constrain available septal landing sites. The need for additional device therapy in the future should be based on extraction feasibility, residual RV anatomy, and alternative pacing modalities.

This report is mainly limited by focus on a single patient, inferential mechanism of oversensing, and incomplete historical interrogation data from the original Micra device. Exact spacing between devices was not measured, and formal artefact frequency analysis was unavailable. Therefore, the findings should be viewed as a practical troubleshooting experience rather than definitive mechanistic proof. From a guideline perspective, the case supports individualized device programming and optimization of sensing and capture parameters while minimizing avoidable procedural risk in vulnerable patients.^2,13^

## Conclusion

Mechanically mediated device–device interaction between sequential Micra leadless pacemakers may cause oversensing and pacing inhibition. Programming optimization may be considered before invasive revision in carefully selected patients with clinically stable status and adequate intrinsic R-wave amplitude. However, this single-case experience should be interpreted cautiously, and future management must account for cumulative RV device burden and the potential need for extraction or alternative pacing strategies.

## Data Availability

All relevant data are included within the article. No additional data are available.

## Lead author biography

As a chief physician, Wenchang Zhang focuses on the field of cardiac electrophysiology and pacing, particularly on the ablation of complex arrhythmias and physiological pacing.

## Notes

**Consent:** In accordance with institutional policy, formal ethics committee approval was not required for this report of a single case. Written informed consent was obtained from the patient for publication of this case report and any potentially identifiable images or data.
